# Genome-wide study of globally distributed respiratory syncytial virus (RSV) strains implicates diversification utilizing phylodynamics and mutational analysis

**DOI:** 10.1038/s41598-023-40760-y

**Published:** 2023-08-19

**Authors:** Tushar Ahmed Shishir, Otun Saha, Sultana Rajia, Spencer Mark Mondol, Md. Habib Ullah Masum, Md. Mizanur Rahaman, Foysal Hossen, Newaz Mohammed Bahadur, Firoz Ahmed, Iftekhar Bin Naser, Mohammad Ruhul Amin

**Affiliations:** 1 Department of Mathematics and Natural Sciences, BRAC University, Dhaka, Bangladesh; 2 Department of Microbiology, Noakhali Science and Technology University, Noakhali, Bangladesh; 3 Department of Microbiology, University of Dhaka, Dhaka, Bangladesh; 4 Department of Chemistry, Noakhali Science and Technology University, Dhaka, Bangladesh

**Keywords:** Computational biology and bioinformatics, Genetics, Microbiology, Pathogenesis

## Abstract

Respiratory syncytial virus (RSV) is a common respiratory pathogen that causes mild cold-like symptoms and severe lower respiratory tract infections, causing hospitalizations in children, the elderly and immunocompromised individuals. Due to genetic variability, this virus causes life-threatening pneumonia and bronchiolitis in young infants. Thus, we examined 3600 whole genome sequences submitted to GISAID by 31 December 2022 to examine the genetic variability of RSV. While RSVA and RSVB coexist throughout RSV seasons, RSVA is more prevalent, fatal, and epidemic-prone in several countries, including the United States, the United Kingdom, Australia, and China. Additionally, the virus's attachment glycoprotein and fusion protein were highly mutated, with RSVA having higher Shannon entropy than RSVB. The genetic makeup of these viruses contributes significantly to their prevalence and epidemic potential. Several strain-specific SNPs co-occurred with specific haplotypes of RSVA and RSVB, followed by different haplotypes of the viruses. RSVA and RSVB have the highest linkage probability at loci T12844A/T3483C and G13959T/C2198T, respectively. The results indicate that specific haplotypes and SNPs may significantly affect their spread. Overall, this analysis presents a promising strategy for tracking the evolving epidemic situation and genetic variants of RSV, which could aid in developing effective control, prophylactic, and treatment strategies.

## Introduction

After being discovered in chimpanzees in 1955, the respiratory syncytial virus (RSV), an RNA virus of the Paramyxoviridae family and Pneumovirus genus, was shortly found to be a pathogen in humans^1^. In the first two years of life, 90% of infants are infected with RSV and at risk of reinfection at any moment. Lower respiratory infections occur in many RSV patients^1^. In children under five, the virus is the leading cause of acute lower respiratory tract disease (LRTI)^2^. About 40% of RSV infections in infants may result in LRTI, mostly pneumonia and bronchiolitis^2^. As a result, up to 199,000 pediatric fatalities, 3 million hospitalizations, and over 33 million LRTIs are all attributed to RSV worldwide^1^. In Canada, RSV causes 5800 to 12,000 hospitalizations annually, with a reported increase in bronchiolitis during the last two decades^2^. Between 1997 and 2000, RSV bronchiolitis accounted for an average of 77,700 yearly hospitalizations in the United States among babies less than one-year-old. The hospitalization rate for people in this age bracket rose 25% between 1997 and 2002^2,3^.

RSV, like influenza viruses, has a seasonal epidemic, peaking between October and December in temperate countries and April and September in tropical and subtropical regions, but with milder symptoms^1,2^. Recent studies in China found 402, 288, and 415 confirmed cases of RSV infections over three consecutive years (2019, 2020, and 2021)^4^. Of the 8,461 confirmed cases of RSV in Denmark in 2022, another research found that 3,417 required emergency hospitalization^5^. However, preterm newborns, patients with underlying cardiac, pulmonary, neurologic, and immunological issues, and the elderly have a far more significant morbidity and mortality risk from this illness^1,6^. The RSV genome is a single-stranded, non-segmented molecule that is 15,191–15,226 nucleotides long and contains 10 functional genes (3′ NS1-NS2-N-P-M-SH-G-F-M2-L) [9]. Each mRNA typically encodes a single essential protein, except for M2, which includes two separate ORFs that slightly overlap and encode M2-1 and M2-2.

Interestingly, two membrane proteins of the RSV genome envelop, one of which helps with host cell attachment and the other with fusion^1^. Based on the genomic versatility, the virus only has one serotype, but it is further subdivided into two strains, RSVA and RSVB^1,7^. Interestingly, RSVA is more common than RSV B and may lead to more severe illness and even death if it causes an infection^8^. However, all RSV genotypes display a range of pathogenicity^8^. Different RSV genotypes can co-epidemic at the same time and place. However, the majority of epidemics are dominated by one of the subgroups or genotypes^14,15^. In China, the ON1 RSVA genotype and the BA9 RSVB genotype have recently been the two most prevalent genotypes^14,16^. This is because; RSV shares the high mutation rate of other RNA viruses (103 to 104). However, only the G gene was confirmed to have mutated in vivo research, despite in vitro evidence suggesting that both the G and SH genes are more susceptible to spontaneous mutations than any other portion of the RSV genome^10,17^. In response to immunological pressure, circulating RSV seems to undergo persistent alterations in sequence and antigenicity, particularly in the G protein^10^. However, duplication in the C-terminal region of the attachment (G) glycoprotein (ON1-like genotype) of RSVA was first discovered in 2010 in Canada^17^. Furthermore, an RSV epidemic was described in Australia during the COVID-19 era of 2020–2021, particularly affecting the ON1 genotype of RSVA, with many point mutations detected in the C-terminal region of the attachment (G) glycoprotein, in addition to the nucleocapsid and tiny hydrophobic proteins^18^. Therefore, mutations to these proteins increase the viral epidemic propensity, drug resistance, and immune tolerance.

Therefore, by considering all of the global issues, we determined to conduct a comprehensive genome study of RSV in which the purpose of this research was to examine the differences in mutations between RSV subtypes A and B, as well as their transmission, phylogenetic, and phylodynamic analyses. We expect this research to be instrumental in developing an effective RSV preventative and addressing severe respiratory conditions such as viral lower respiratory tract infections worldwide.

## Results and discussion

### Phylogenetic and transmission network analysis

**Figure 1:**
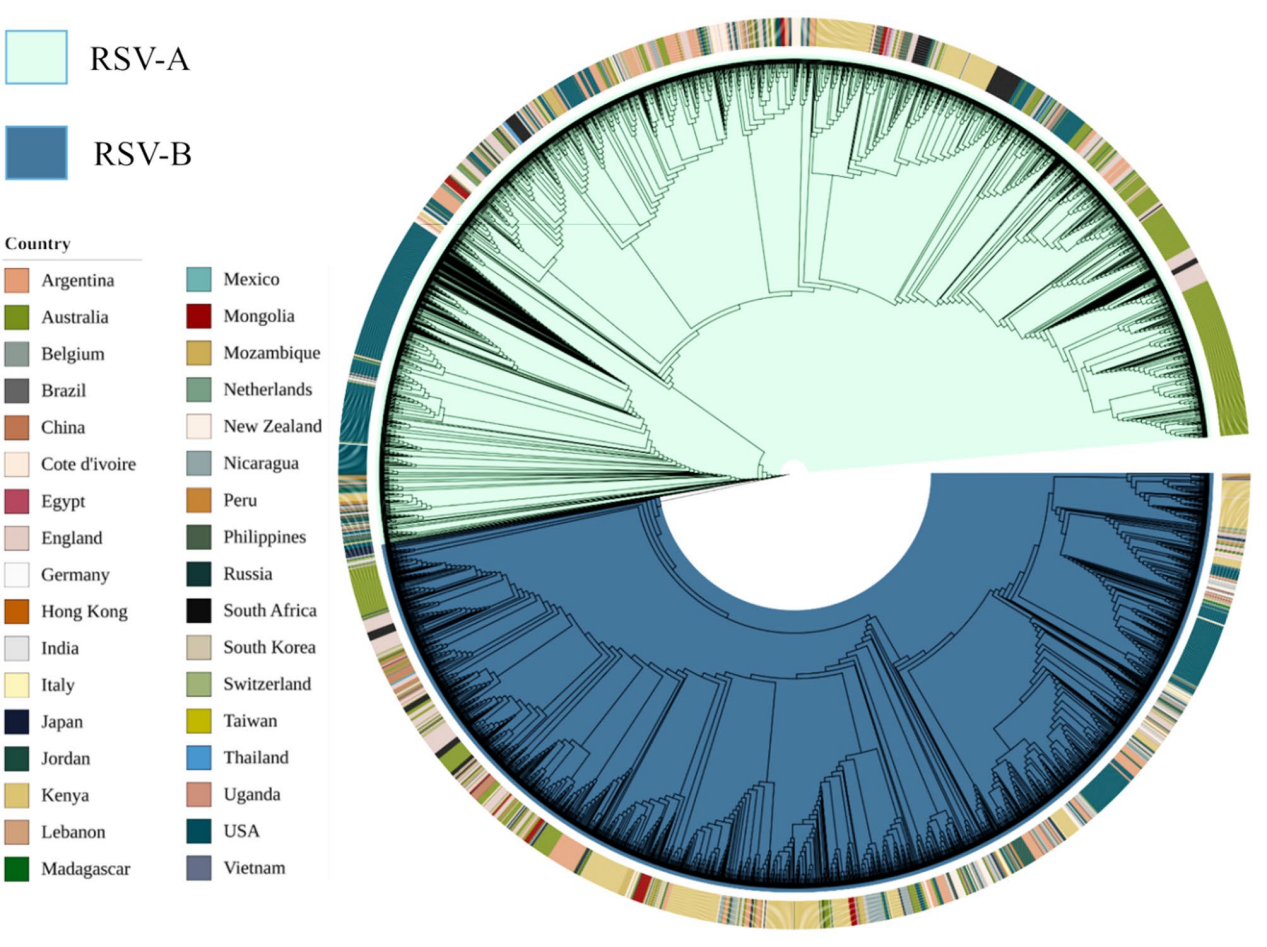
A spherical, combined representation of the geographic ranges and evolutionary trees of RSV A and RSV B. The perimeter of the circle is displayed with distinct colors, one for each country, to show the geographical distribution of these two strains.

**Figure 2:**
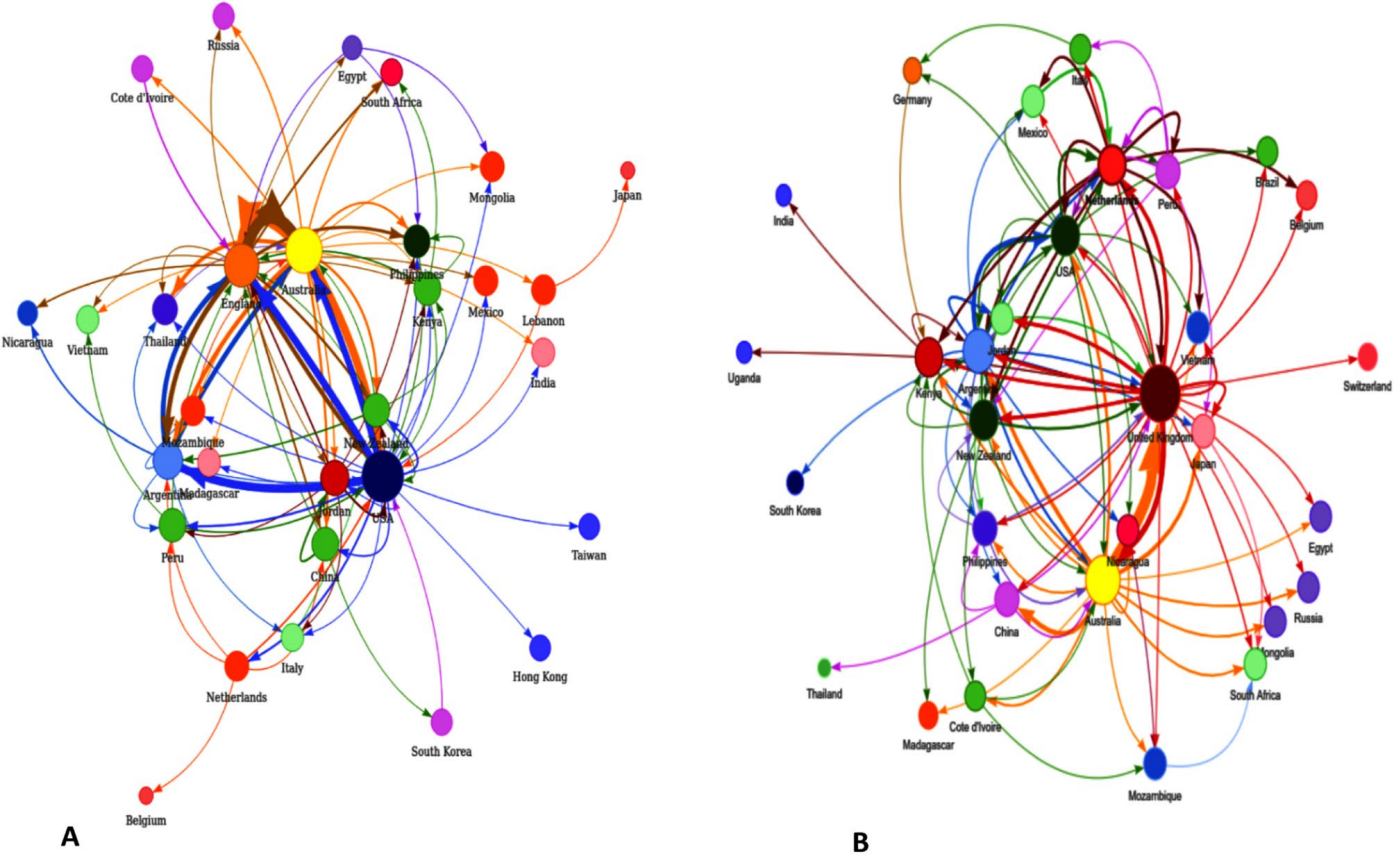
Both RSVA (**A**) and RSVB (**B**) have transmission networks that can be predicted using StrainHub. Direction of transmission is shown by arrows, while nations of origin are denoted by circles of varying colours.

Phylogenetic and transmission network analyses provide significant insight into the global evolution, geographical spread, and transmission dynamics of RSV-A and RSV-B. We extensively analyzed the evolutionary history of RSV worldwide by utilizing phylogenetic and phylodynamic analyses^38^ and estimated that RSV-A and RSV-B appeared around 1953 and 1956, respectively. In addition, we observed significant variations in the virus circulating in various countries at different times. Overall, RSV-A was present in a broader number of countries than RSV-B (Fig. 1). Transmission analysis showed that the USA, the UK, and Australia served as the central nodes for both RSV-A and RSV-B transmission (Fig. 2). Considering the high number of international visitors to these three countries, there exists a greater possibility of cross-border transmission of infectious diseases. Our findings indicated that RSV-A spread from the UK and Australia to Russia but could not spread beyond (Fig. 2A). These three countries served as a conduit for the virus to spread to other countries. The transmission network analysis also revealed that RSV-A was acquired by the Netherlands from the USA and spread to China, Peru, Belgium, and finally to the USA. In contrast, strain B spread to the Netherlands through many countries, including the USA, the UK, Australia, Peru, Mexico, and Argentina. Figure 2B illustrates that Argentina played a key role in the spread of both strains throughout South America. Then, India acquired RSV-A from the USA and Australia and imported RSV-B from the UK, Australia, New Zealand, Cote d'Ivoire, Germany, and the Netherlands. There was, however, no transmission of infectious strains outside of India was observed which was surprising since. India is ranked second most populated country in Asia and seventh in the world.

It was anticipated that China, the Philippines, and Vietnam would be the Asian countries with the highest transmission rates for both strains. China was infected with RSV-A from the USA, Australia, Great Britain, and the Netherlands, but not with strain B. Thailand had the highest transmission rate for RSV-A, whereas Japan had the highest transmission rate for RSV-B. Several other nations, including Mexico, Peru, Belgium, and Vietnam, have also been identified as major transmission centers for strain B. Furthermore, RSV A and RSV B were imported to Russia from the United Kingdom and Australia, two highly populated countries; however, no evidence was found to indicate the virus had spread outside Russia , maybe due to lack of viral sequences. As a whole, the transmission network showed that RSV-A and RSV-B had quite different transmission patterns outside of major transmission centers like the USA, the UK, and Australia, consistent with previous studies^8,13^. Globalization, climate change, seasonal shifts, and travel between countries are some potential causes of the widespread transmission pattern in these countries.

### Haplotype analysis of RSV

**Figure 3:**
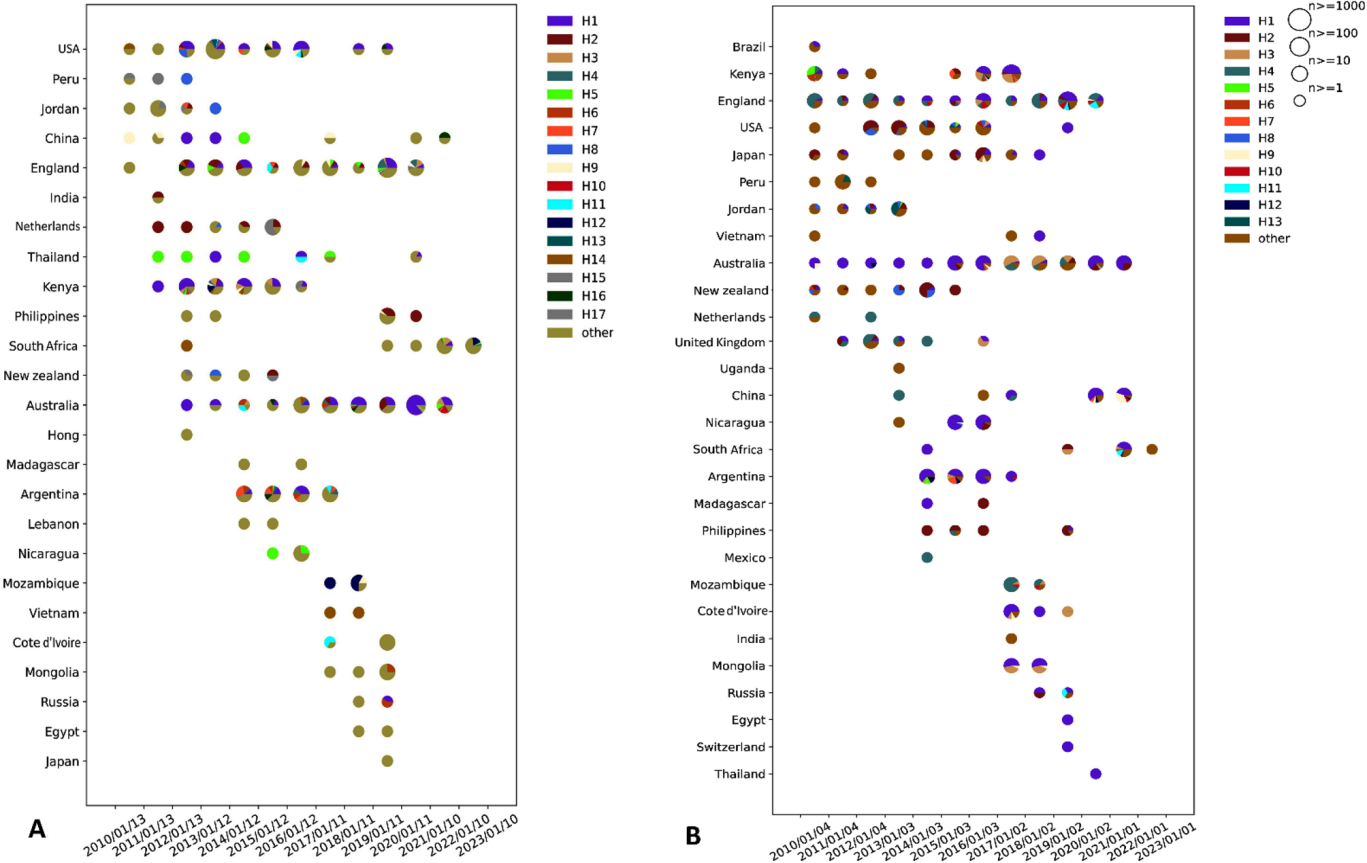
The worldwide spread of 13 more haplotypes of respiratory syncytial virus (RSV) (**A**) and (RSVB) (**B**). The size and color of the circles stand in for the total size of the genome and the total number of haplotypes, respectively.

Researchers referred to the several sets of mutations or variations detected in a particular region of the virus's genomes as haplotypes^14,17^. The haplotype analysis of all strains indicated that several haplotypes may develop in different countries throughout time^42^. Therefore, research on virus haplotypes is essential for identifying genetic markers associated with specific virus strains, developing effective diagnostic tools, vaccines, and therapies, tracking viral spread and predicting outbreaks, as well as monitoring the emergence of new strains. In light of this, we analyzed the genomes of the RSV viruses and discovered multiple haplotypes that emerged over time in different countries. For example, the H5 haplotype was Thailand's most predominant RSV A haplotype until 2012/2013, as depicted in Fig. 3A. Thailand was the first country to encounter this haplotype in 2010–2011. However, it was found to be declining when the H1 haplogroup became more prevalent around 2013. Nevertheless, this haplogroup was found to appear again in 2014. Interestingly, around that time, England (between 2013 and 2014) and China (since 2014) were identified were identified to carry the haplogroup H5, so it likely returned to Thailand in 2014, either from England or from China. Before 2010, China possessed a large percentage of the H9 haplotype, the world's biggest country. The H5 and H1 haplogroups were most common there in 2012 and afterwards, but after 2018, the H9 haplotype emerged again. China was found to carry the H16 haplotype in 2021–2022, which was first discovered in England in 2012–2013. This haplogroup originated in England and spread to the United States, where it circulated for a while, then to Australia and Argentina. It also probably entered China in late 2020 from the United States.

In America, Japan, the Philippines, and New Zealand (Fig. 3B), H2 was the most prevalent haplotype of RSV B. The H2 haplotype was first identified in Japan, Kenya, and New Zealand in 2010 and 2011 and then appeared in the United Kingdom and the United States in 2012. The H2 haplotype reached its maximum prevalence in the US between 2012 and 2013 and declined afterward. The H2 haplotype was not discovered in Australia until 2013. As soon as the H2 haplotype first appeared in Australia in 2014, it reached New Zealand and became the most prevalent there in 2014 and 2015. The H1 haplotype has been the most prevalent in Australia in the following years. After 2017, the H3 haplotype became more prevalent in Australia, while the H1 haplotype declined. In 2016, H3 was detected in Kenya, England, the United States, and Japan. The H3 haplotype likely arrived in Australia from one of these regions. By 2021, the H11 was the most common haplotype in Australia. H11 was first discovered in Philippines in 2014 and in Japan in 2015. As a result of the proximity between Japan and the Philippines, this haplotype could have been introduced from the Philippines to Japan. In contrast, the H11 haplotype moved to England the following year after its exposure in Kenya, remaining there until 2021. In 2021, it was found in South Africa. Thus, the H11 haplotype of RSVB is thought to have traveled from Kenya to England and South Africa.

However, it is important to note that while our analysis provides insights into the evolution of RSV haplotypes, our predictions are based on the available data, which vary depending on the degree of sequence data quality in different regions in different regions as well as the number of sequences. Overall, our study highlights the dynamic nature of RSV haplotypes and their global spread, which may have significant implications for vaccine and treatment development.

### Mutational analysis

The molecular evolution of respiratory syncytial virus (RSV) has been demonstrated to be significantly influenced by mutation and recombination in previous studies. RSVA's genetic and phenotypic evolution is primarily driven by selection dynamics^39,40^. Therefore, mutational analysis was conducted on RSVA and RSVB, which revealed all possible mutations, including blank, missense, insertion, deletion, frameshift, and stop codon mutations (Table 1).

**Table 1:** Summary of all calculated mutations in RSV A and RSV B by SNP-sites.

Mutations	Strain	
	RSVA	RSVB
Root Date	1953	1956
Mutation Rate	0.000855	0.000777
Total Unique Mutations	13,408	10,922
Unique Synonymous Mutations	8540	6638
Unique Missense Mutations	4813	4182
Unique Insertion Mutations	14	44
Unique Deletion Mutations	21	20
Unique Stop Codons Gained	20	38

*RSVA* respiratory syncytial virus strain A, *RSVB* respiratory syncytial virus strain B.

In our study, we found that the genomes of RSV A and RSV B contain a high number of unique mutations. There were 13,408 unique mutations in RSV A, including 8,540 synonymous mutations and 4,813 missense mutations. RSV B had a total of 10,922 unique mutations, including 6,638 synonymous mutations and 4,182 missense mutations (Supplementary Fig. 1).

Interestingly, both RSV A and RSV B had a roughly the same percentage of missense mutations, with RSV A having a percentage of 35.85% and RSV B having a percentage of 38.23%. In contrast, RSV A had a higher mutation density per base than RSV B, with 0.00135 mutations per base compared with 0.00124 mutations per base for RSV B. While RSV A had a greater number of synonymous, missense, and deletion mutations, RSVB had 44 insertion mutations, an increased number than RSVA's total of 14.

**Figure 4:**
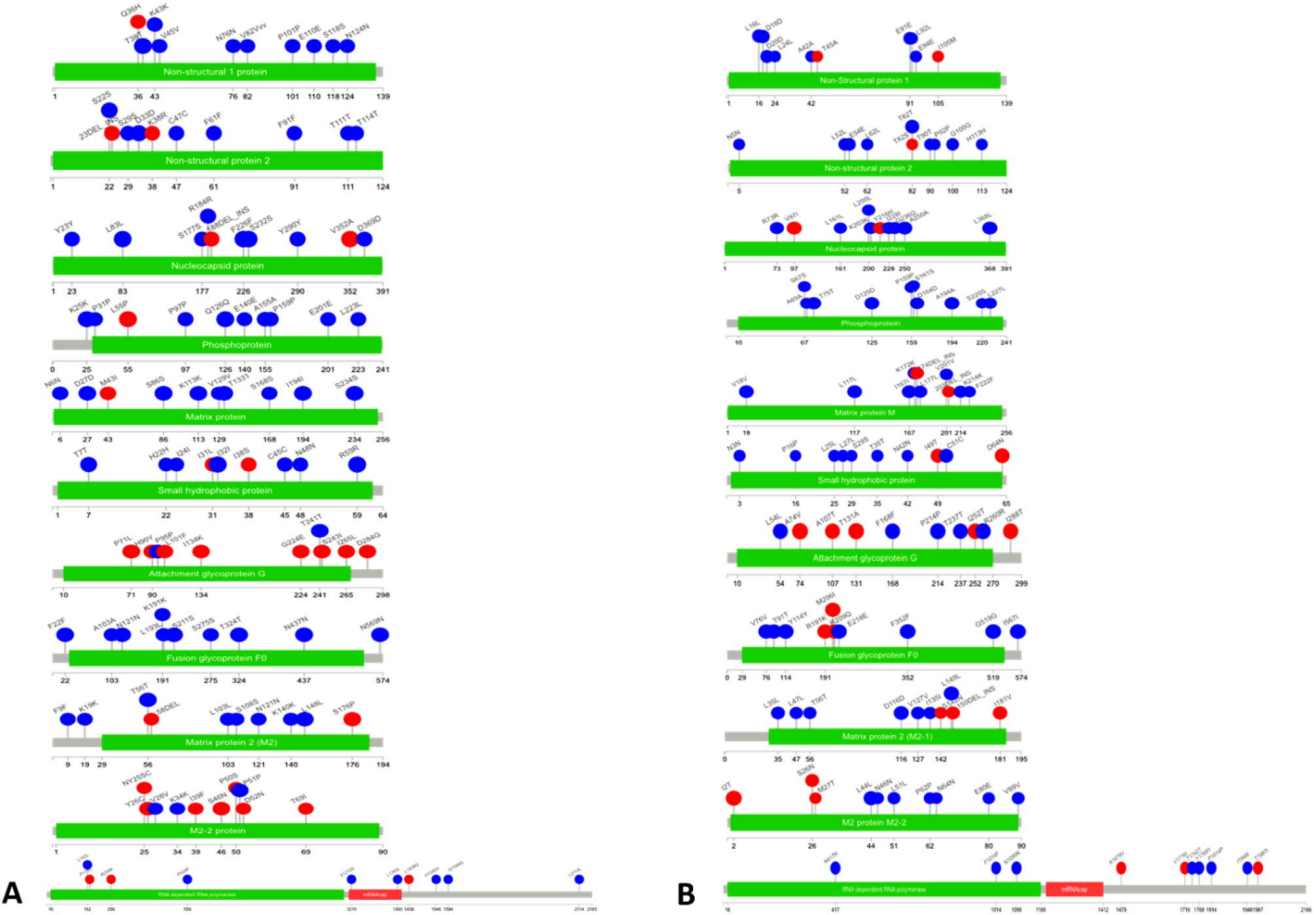
The lollipop plot represents all possible mutations in RSV A (**A**) and RSV B (**B**). Each gene is represented by the green bar, and each mutation within that gene is shown on the lollipop as a single-letter change in the coding for an amino acid. Lollipops in blue represent synonymous mutations while those in red represent missense mutations.

Attachment glycoproteins showed a higher mutation density per base than any other protein for both strains^39^, also harbored the most prevalent mutations which indicates a greater greater diversity (Fig. 4A and B). In RSV A, this protein had the greatest number of missense mutations (69.25%), followed by the M2-2 protein (67.5%), whereas RSV B had a greater percentage of missense mutations in the M2-2 protein and the small hydrophobic protein compared to RSV A. A total of 1229 missense were found in the polymerase protein of RSVB, while 1,494 missense mutations were found in the attachment glycoprotein. Both strains had the highest mutation density per base for attachment glycoproteins (Tables 1, 2, 3, and Supplementary Fig. 1), while the lowest mutation density per base was found for nucleoproteins.

**Table 2:** The detailed and individual mutational analysis of RSV A genome.

Gene	No. of synonymous mutations	No. of missense mutations	Percentage of missense mutations (%)	Mutation density per base	No. of insertion mutation	No. of deletion mutation	No. of stop codon gained
Attachment glycoprotein	678	1527	69.25	2.46	1	3	14
Fusion glycoprotein	1180	498	29.68	0.97	1	1	0
M2-1 protein	354	154	30.31	0.87	0	1	0
M2-2 protein	131	272	67.49	1.48	2	0	2
Matrix protein	547	114	17.25	0.86	0	5	0
Nonstructural protein 1	335	90	21.18	1.01	1	1	0
Nonstructural protein 2	276	124	31.00	1.07	0	0	0
Nucleoprotein	699	138	16.49	0.71	1	1	0
Phosphoprotein	457	165	26.53	0.86	1	1	0
Polymerase Protein	3704	1625	30.49	0.82	7	8	3
Small Hydrophobic Protein	179	106	37.19	1.46	0	0	1

**Table 3:** The detailed and individual mutational analysis of RSVB genome.

Gene	No. of synonymous mutations	No. of missense mutations	Percentage of missense mutations	Mutation density per base	No. of insertion mutation	No. of deletion mutation	No. of stop codon gained
Attachment glycoprotein	693	1494	68.31%	2.43	22	4	20
Fusion glycoprotein	887	422	32.24%	0.76	2	3	0
M2-1 protein	259	119	31.48%	0.64	0	0	2
M2-2 protein	88	155	63.79%	0.89	1	0	0
Matrix protein	394	71	15.27%	0.60	0	1	1
Nonstructural protein 1	269	107	28.46%	0.90	1	1	2
Nonstructural protein 2	200	142	41.52%	0.91	1	0	0
Nucleoprotein	532	126	19.15%	0.56	2	0	0
Phosphoprotein	365	166	31.26%	0.73	1	2	1
Polymerase Protein	2818	1229	30.37%	0.62	14	9	9
Small Hydrophobic Protein	133	151	53.17%	1.43	0	0	3

An overview of the mutational report for RSVA and RSVB, including the frequency and the most recent occurrence of each mutation and the originating and terminating countries for each mutation, can be found in supplemental Tables 1 and 2. This meta-data can be utilized by researchers to better comprehend the potential consequences of mutations on the virus's pandemic potential. Overall, the mutational analysis of RSVA and RSVB provides valuable insights into RSV evolution, particularly the attachment glycoprotein's role in determining mutational dynamics. The information provided here can assist in the development of more efficient diagnostic and therapeutic approaches for RSV infections.

### Linkage disequilibrium (LD) interpretation

There is a degree of association between significant mutations and their predicted relationships known as linkage disequilibrium (LD). We performed LD analysis to evaluate the degree of genetic linkage between single nucleotide polymorphisms (SNPs) and haplotypes. Our analysis indicated that there was a low level of genetic linkage between SNPs and haplotypes (Supplementary Fig. 2).

**Figure 5:**
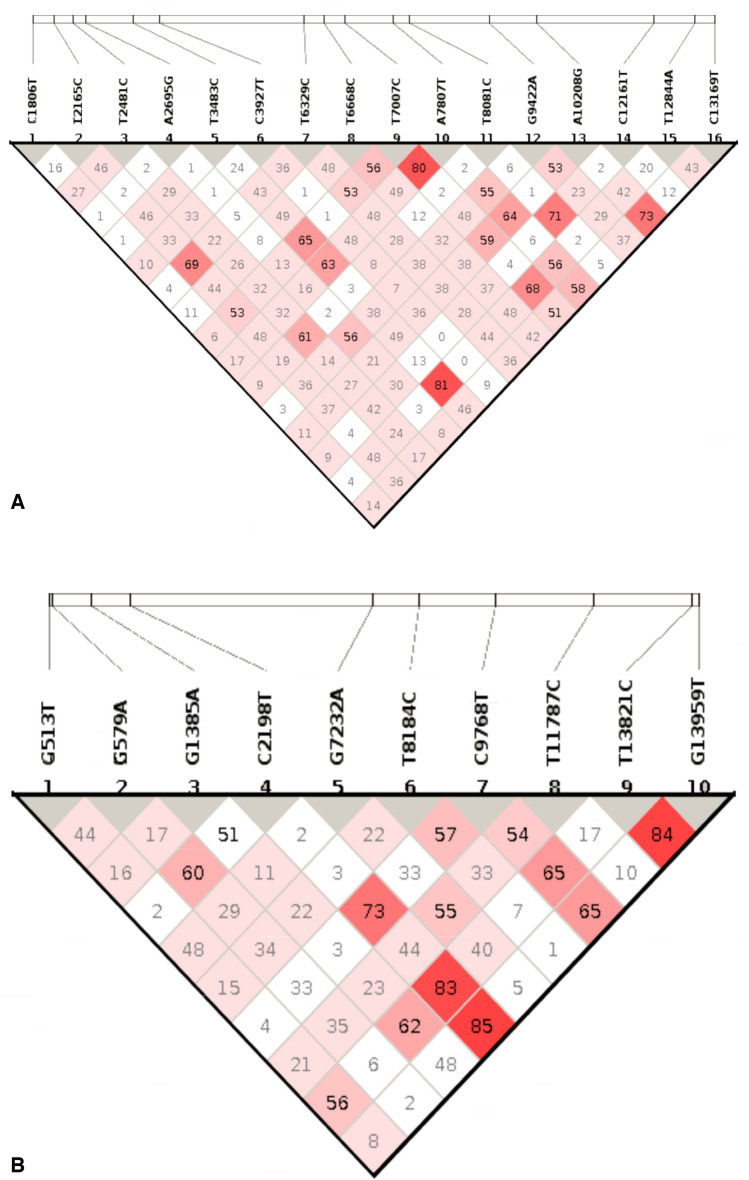
Haploview's determination of LDs between SNPs in RSV A (**A**) and RSV B (**A**) is shown in the haplotype block. As the association between the LDs grows with colour, from white (the weakest) to crimson (the highest), the colour serves as a reflection of the LDs' true powers. The bases of the genome that have been changed due to mutations are marked in the text at the top of the picture.

In previous studies^21^, it was identified that loci T12844A and T3483C had the highest probability of linkage, and we also found a correlation between these loci, with an R2 value of 0.81, demonstrating that both mutations occurred simultaneously in 81% of cases. In addition, the SNPs A7807T and T7007C had the second-highest linkage probability with an R2 of 0.80. One in eight individuals is likely to identify these two mutations simultaneously (Fig. 5A)^25^. In terms of evolutionary and fitness perspectives^43,44^, these genetic linkages between mutations must be of significant importance. Nevertheless, the most likely pair in RSV B is G13959T and C2198T with an R2 value of 0.85, and T13821C has a genetic relation with G13959T with an R2 value of 0.84. It is possible that these two SNPs are related if they occur at the same location. Previous research demonstrated a significant association or relationship between C2198T and another SNP, T13821C. On this basis, we suppose a significant connection exists between the co-occurrence of these three SNPs in strain B (Fig. 5B).

Overall, a number of key insights were found when we examined the degree of association between significant mutations and their genetic linkage, suggesting that certain genetic variants will likely evolve if these mutations persist. Further, several regions of high LD suggest that the viral genome will likely contain haplotype blocks inherited with certain combinations of genetic variants, indicating a high genetic structure. A highly structured genome and a group of haplotypes in the RSV virus suggest a complex genetic architecture that may play a role in the same trait and evolution of a new variant. Therefore, these findings have profound implications for understanding the pathogenesis of RSV infection. Further diagnostic tests and treatment strategies could be developed based on the identified loci that are likely to play a role in the infection process. Lastly, it is also possible to develop personalized and targeted interventions for the RSV virus infection by identifying specific genetic variants associated with disease susceptibility or severity.

### Effects of mutations on epidemic nature and evolutionary dynamics

On the basis of our analysis, the nucleotide diversity of the attachment glycoprotein of RSV-A was the highest, with a value of 0.0682, followed by the fusion glycoprotein and nucleoprotein. On the other hand, the M2-1 protein had the lowest nucleotide diversity (0.00571). Nucleotide diversity in populations may change due to natural selection. Further, Shannon's entropy values for all the genes were relatively high, ranging from 0.307252 for the phosphoprotein to 0.673744 for the attachment glycoprotein, indicating a high degree of heterogeneity within the genes (Supplementary Fig. 3A). All Tajima's D values were negative, ranging from -2.74577 for the matrix protein to − 0.158127 for the M2-1 protein, suggesting that the RSV-A population has recently expanded or that positive selection has been exerted on these genes. The dN/dS ratio ranges from 0.0492 for the nucleoprotein to 0.555 for the attachment glycoprotein, indicating varying degrees of positive selection. The attachment glycoprotein gene had 30 sites towards positive selection, followed by polymerase protein. In contrast, the polymerase protein gene had 1028 sites towards negative selection, followed by nucleoprotein and attachment glycoprotein (Supplementary table 1).

For RSV-B, the gene with the highest nucleotide diversity (π) was the attachment glycoprotein with a value of 0.02861, followed by the polymerase protein and the fusion glycoprotein, while the lowest diversity was found in the gene of M-2 protein with a value of 0.00533. A high degree of diversity exists within the gene sequences as evidenced by Shannon's entropy values, which ranged from 0.272656 for nonstructural protein 1 to 0.953729 for small hydrophobic protein (Supplementary Fig. 3B). There were negative Tajima's D values for all the genes, ranging from -2.76488 for the M2-2 protein to -2.20653 for the attachment glycoprotein. This suggests that the RSV-B population has expanded or genes have been subject to positive selection. The dN/dS values were below 1 for all genes, ranging from 0.049 for the nucleoprotein to 0.506 for the attachment glycoprotein. This indicates that genes have been subject to negative selection to maintain their function. In terms of positive selection, the polymerase protein gene had the highest number of sites, followed by the fusion glycoprotein and the attachment glycoprotein genes. A higher number of sites toward negative selection were observed in the polymerase protein gene, followed by the nucleoprotein and the attachment glycoprotein (Supplementary table 2). The number of sites towards positive selection in RSV B is significantly lower than that of RSV A, with the polymerase protein having just five sites toward positive selection. In contrast, the number of negative selection sites ranges from 16 for a small hydrophobic protein to 928 for a polymerase protein.

Additionally, RSV A exhibited greater nucleotide diversity than RSV B. However, genetic variability was not uniform across all genes. As evident from the results of both viruses, the attachment glycoprotein gene exhibited the greatest nucleotide diversity and the greatest number of positive selection sites, suggesting that this gene has evolved and adapted to the host environment under strong positive selection pressure. However, the Nucleotide diversity was three times higher in RSV-A's attachment glycoprotein than RSV-B, suggesting a higher mutation rate and perhaps more mutations, which might have implications for vaccine development. Moreover, the polymerase gene also displayed a high number of sites towards positive selection in both viruses, indicating that it plays an important role in viral replication and evolution. In addition, the attachment glycoprotein demonstrates the highest dN/dS ratio in both subtypes, with a negative Tajima's D value, indicating that the gene may be evolving drastically or the virus population is expanding because there are more low-frequency polymorphisms than predicted. There might be a variety of reasons for this. Recent population expansion may be responsible for an increased ratio of rare to common mutations^51,52^. Another possibility is selective sweeps, when a beneficial mutation rapidly spreads across a population, wiping out variants in the process^53^. Further research is needed to determine if the occurrence of a negative Tajima's D value is the consequence of demographic events, selection pressures, or some other cause^53^. The data showed that the favored trend was negative selection. In general, it is clear from the data that both subtypes of RSV are subject to different selective pressures and have unique mutation profiles, which could affect the development of a vaccine and treatment.

## Conclusion

RSV A and RSV B were studied comprehensively by phylogenetic analysis, phylodynamic modeling, and mutational analysis to understand how mutations evolved, were transmitted, and selected. We have found that highly mutated surface attachment glycoprotein and fusion protein would make developing effective RSV A and B vaccines challenging. RSV A was found to have a higher mutation rate than RSV B, and transmission networks, SNPs, and Shannon's entropy differed significantly. To sum up, our study has significant importance and applications in developing respiratory syncytial virus (RSV) therapeutics and diagnostics. Understanding RSV's genetic variability is important to develop effective diagnostic tools and treatment strategies. An extensive mutational study can be used to identify a drug target or vaccine epitope targeting structural proteins against the virus. Linkage distribution data could be incorporated along with mutational data in order to gain insights about haplotypes and develop targeted antiviral therapies to increase their effectiveness. Further, in-silico drug design approaches could be used to develop antiviral compounds specifically targeting critical regions of the virus. The data can be incorporated into ongoing surveillance systems to monitor the emergence of new strains and potential outbreaks, assist public health authorities in planning and responding promptly, and develop diagnostic tools. Overall, our research findings will help develop therapeutic and diagnostic drugs, aiding in identifying drug targets, in-silico drug design, rapid diagnostic tests, and tracking viral variants for effective control and treatment.

## Materials and methods

### Sequence retrieval

The Global Initiative for Sharing All Influenza Data (GISAID) has been searched for all submitted Respiratory syncytial virus subtype A and B genomic sequences as of December31, 2022^19^. All of the sequences were then screened for quality before further processing, and those with gaps were thrown out. According to GISAID, the EPI_ISL_412866 and EPI_ISL_1653999 sequences were chosen as reference sequences for the RSV A and B subtypes, respectively.

### Phylogenetic and transmission analysis of RSV genomes

Firstly, the selected sequences were aligned using the Mafft algorithm^20^, followed by the construction of a maximum likelihood phylogenetic tree using the IQ-TREE tool with a bootstrap value of 1000^21^. After that, we reconstructed the tree using the TreeTime tool^22^ so that we could have a clear understanding of the chronological relationship. We then annotated the reconstructed tree with geography information and visualized it using the iTOL server^23^. Based on the same procedure, we constructed phylogenetic trees for both RSV A and RSV B, which were then used to construct their transmission network using StrainHub^24^. Lastly, we classified the sequences into different haplotype groups and analyzed their chronological distribution over time at different geographical locations as well as their back-and-forth movement in different parts of the world using the AutoVem2 tool^25^.

### Mutation analysis

The minimap2 algorithm^26^ was used to align all the sequences of RSV A and RSV B against the relevant references, and Samtools was used to call the variants from the alignments. Further, SNP-sites^27^ were also used to detect mutations in the sequences, and only common mutations were analyzed downstream. Afterwards, SNPeff^28^ was used to predict the effects of the mutations. Haploview^29^ was used to detect linkage disequilibrium among the mutations with the highest prevalence and presented as R2 index, whereas the lollipops tool was used to prepare the lollipop plots of those mutations. In general, R programming was used for data preparation and analysis.

### Effects of mutation on genome fitness

First, we used TASSEL software^30^ to estimate nucleotide diversity and Tajima's D (π) using a 20 base-pair window at five base-pair steps. In addition, Shannon's Entropy was calculated using DiMA^31^. In the next step, we analyzed the direction of selection in the sequences to determine whether diversity moves away from neutrality and to understand the pattern of evolution using the SLAC algorithm^32^ in the HyPhy software^33^. As a final step, FEL^32^ and FUBAR^34^ methods were employed to identify specific sites that were experiencing diversifying or purifying selection.

### Supplementary Information

Supplementary Information.

## Data Availability

This study was carried out with data retrieved from GISAID database (https://www.gisaid.org/). All data used is available in public databases.
