# Supplementary material for: Genome-wide study of globally distributed respiratory syncytial virus (RSV) strains implicates diversification utilizing phylodynamics and mutational analysis
Source: Sci Rep. 2023 Aug 19;13:13531. doi: 10.1038/s41598-023-40760-y (PMC10439963; doi:10.1038/s41598-023-40760-y)
Supplement: Supplementary file 1 — Supplementary Information. [file 41598_2023_40760_MOESM1_ESM.docx]

**Genome-wide Study of Globally Distributed Respiratory Syncytial Virus (RSV) Strains Implicates Diversification Utilizing Phylodynamics and Mutational Analysis**

**Tushar Ahmed Shishir^1*^, Otun Saha^2*#^, Sultana Rajia^2^, Spencer Mark Mondol^3^, Md. Habib Ullah Masum^2^, Md Mizanur Rahaman^3^, Foysal Hossen^2^, Newaz Mohammed Bahadur^4^, Firoz Ahmed^2^, Iftekhar Bin Naser^1^, Mohammad Ruhul Amin^2#^**

^1^Department of Mathematics and Natural Sciences, BRAC University, Bangladesh.

^2^Department of Microbiology, Noakhali Science and Technology University, Noakhali, Bangladesh.

^3^Department of Microbiology, University of Dhaka, Dhaka, Bangladesh.

^4^Department of Chemistry, Noakhali Science and Technology University, Bangladesh

**#Corresponding Author**

Mohammad Ruhul Amin

Associate Professor

Department of Microbiology

Noakhali Science and Technology University, Noakhali, Bangladesh

E-mail: ruhulmicro4355@gmail.com

and

Dr. Otun Saha

Assistant Professor

Department of Microbiology

Noakhali Science and Technology University, Noakhali, Bangladesh

Mobile: +8801725830666

E-mail: [otun.saha@nstu.edu.bd](mailto:otun.saha@nstu.edu.bd)

*Equal Contribution

#Correspondence


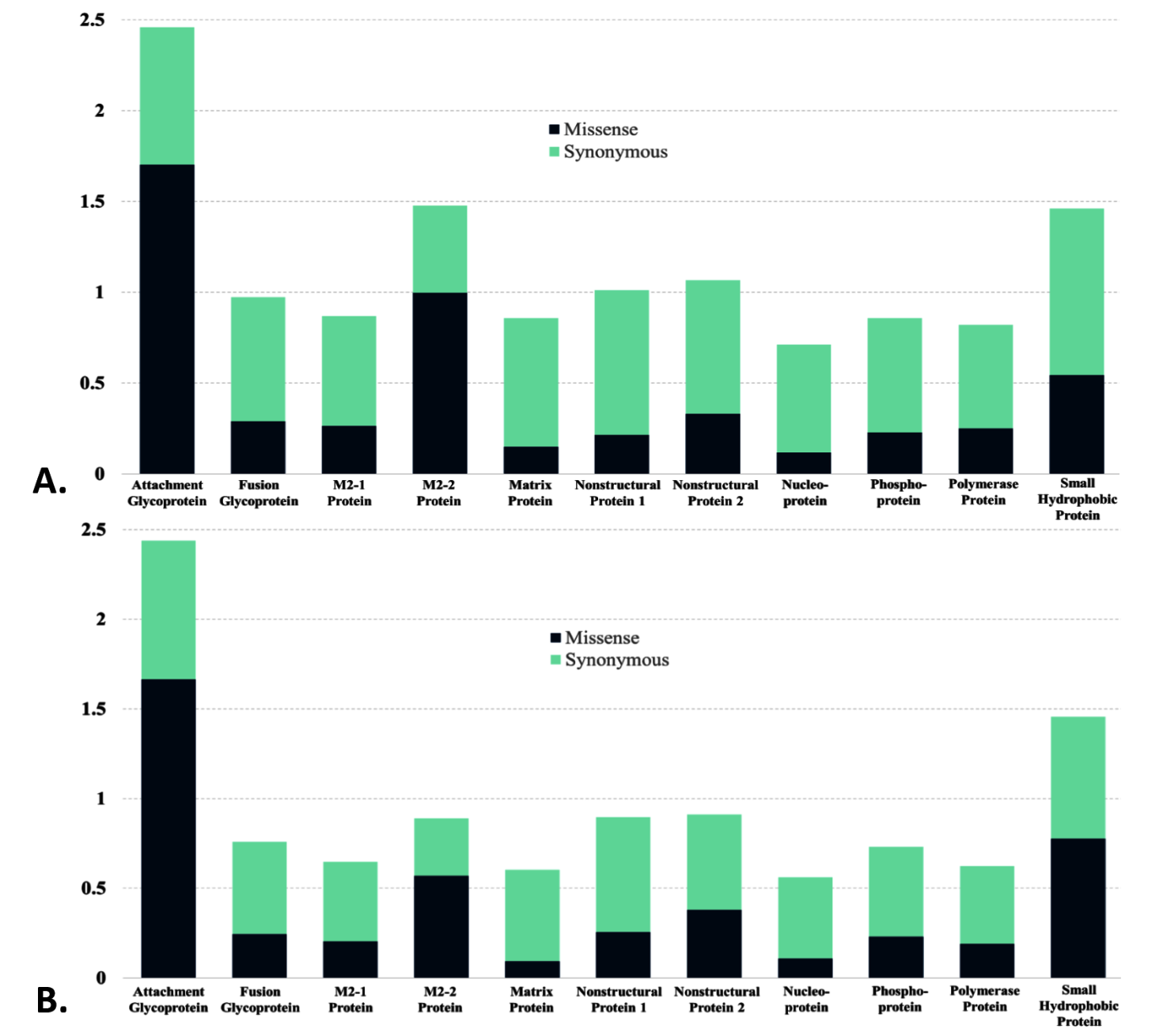


**Supplementary Figure 1.** The mutation per base including missense and synonymous mutation of the proteins from RSV A **(A)** and RSV B **(B)**.


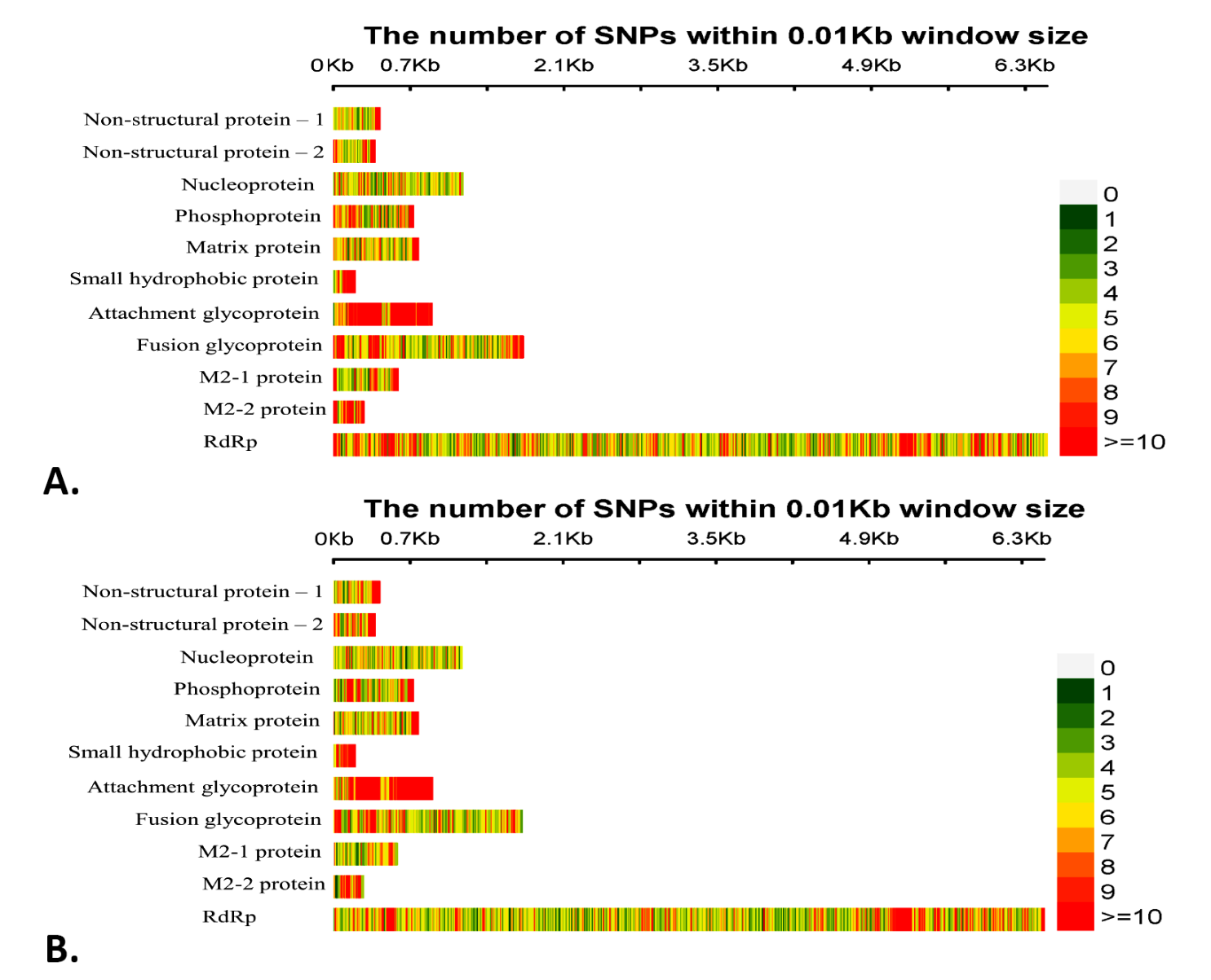


**Supplementary figure 2.** The number of SNPs calculated in RSV A **(A)** and RSV B **(B)** genome. The vertical plot represents the genome size and the color gradient represents the intensity of the SNPs, where red (>10) and white color (0) signifies highest and lowest intensity of SNPs, respectively.


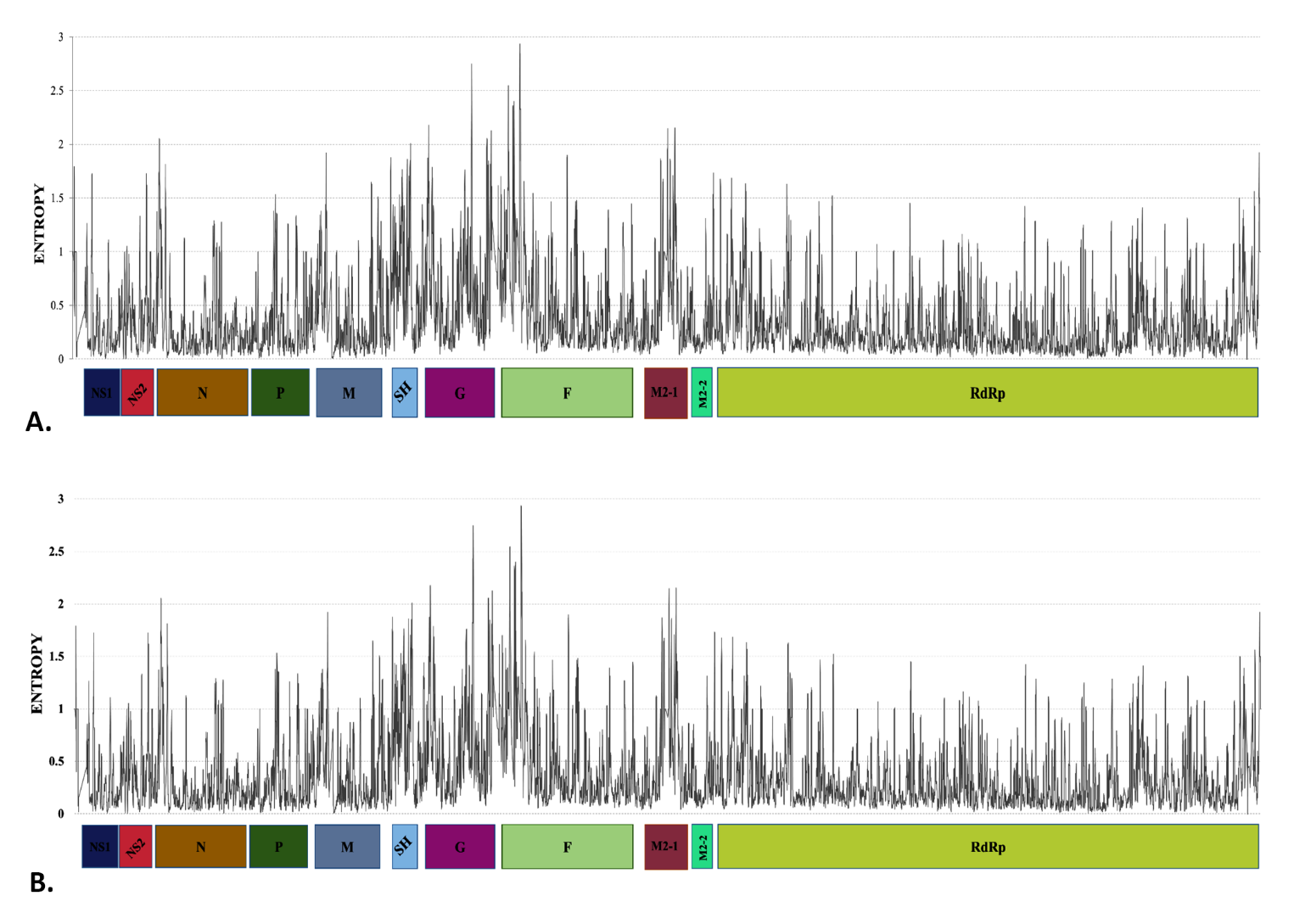


**Supplementary figure 3.** Graphical depiction of Shanon’s Entropy of each protein from RSV A **(A)** and RSV B **(B)** strain.

**Supplementary table 1.** The mutational effects of RSV A including nucleotide diversity, shanon’s entropy, tajima’s D, dN/dS, and the no. of sites towards positive and negative selection.

| Gene | Nucleotide diversity (π) | Shanon’s Entropy | Tajima’s D | dN/dS | No. of sites towards positive selection | No. of sites towards negative selection |
| --- | --- | --- | --- | --- | --- | --- |
| Attachment glycoprotein | 0.0682 | 0.673744 | -1.20544 | 0.555 | 30 | 88 |
| Fusion glycoprotein | 0.05493 | 0.597168 | -1.58848 | 0.126 | 3 | 287 |
| M2-1 protein | 0.00571 | 0.607837 | -2.74577 | 0.146 | 0 | 81 |
| M2-2 protein | 0.04326 | 0.393619 | -1.87467 | 0.44 | 1 | 20 |
| Matrix protein | 0.05086 | 0.402197 | -0.158127 | 0.054 | 0 | 136 |
| Nonstructural protein 1 | 0.01642 | 0.348631 | -2.1172 | 0.112 | 0 | 46 |
| Nonstructural protein 2 | 0.03293 | 0.466468 | -2.07846 | 0.147 | 1 | 55 |
| Nucleoprotein | 0.04528 | 0.407658 | -1.7071 | 0.0492 | 0 | 211 |
| Phosphoprotein | 0.03824 | 0.307252 | -1.83539 | 0.106 | 2 | 124 |
| Polymerase Protein | 0.02071 | 0.391668 | -2.39656 | 0.116 | 7 | 1028 |
| Small Hydrophobic Protein | 0.0411 | 0.595248 | -1.86818 | 0.23 | 1 | 17 |

**Supplementary table 2.** The mutational effects of RSV B including nucleotide diversity, shanon’s entropy, tajima’s D, dN/dS, and the no. of sites towards positive and negative selection.

| Gene | Nucleotide diversity (π) | Shanon’s Entropy | Tajima’s D | dN/dS | No. of sites towards positive selection | No. of sites towards negative selection |
| --- | --- | --- | --- | --- | --- | --- |
| Attachment glycoprotein | 0.02861 | 0.585509 | -2.20653 | 0.506 | 4 | 42 |
| Fusion glycoprotein | 0.02307 | 0.475052 | -2.35027 | 0.133 | 4 | 261 |
| M2-1 protein | 0.01681 | 0.479818 | -2.42557 | 0.167 | 1 | 87 |
| M2-2 protein | 0.00533 | 0.294856 | -2.76488 | 0.412 | 3 | 20 |
| Matrix protein | 0.0211 | 0.334778 | -2.3569 | 0.0528 | 0 | 135 |
| Nonstructural protein 1 | 0.01302 | 0.272656 | -2.41123 | 0.15 | 0 | 47 |
| Nonstructural protein 2 | 0.01155 | 0.374362 | -2.61719 | 0.248 | 1 | 55 |
| Nucleoprotein | 0.0147 | 0.294509 | -2.53579 | 0.049 | 0 | 225 |
| Phosphoprotein | 0.01688 | 0.317647 | -2.44577 | 0.129 | 0 | 126 |
| Polymerase Protein | 0.02597 | 0.308025 | -2.30037 | 0.113 | 5 | 928 |
| Small Hydrophobic Protein | 0.01147 | 0.953729 | -2.62595 | 0.351 | 1 | 16 |
